# Statement of Retraction: Ephedrine alleviates middle cerebral artery occlusion-induced neurological deficits and hippocampal neuronal damage in rats by activating PI3K/AKT signaling pathway

**DOI:** 10.1080/21655979.2025.2515739

**Published:** 2025-06-25

We, the Editor and Publisher of *Bioengineered*, have retracted the following article:

Huang, L., Zhao, B., Li, Q., Wu, J., Jiang, H., & Li, Q. (2021). Ephedrine alleviates middle cerebral artery occlusion-induced neurological deficits and hippocampal neuronal damage in rats by activating PI3K/AKT signaling pathway. *Bioengineered, 12*(1), 4136–4149. https://doi.org/10.1080/21655979.2021.1953218

Following publication, concerns were raised by a third party in December 2024 regarding apparent overlap in Figure(s) 2C, 3A and 5H. An additional concern was raised regarding apparent reuse of figures in other publications:

● Figure 3B appears to be reused as Figure 5 B in:   

Gao H, Peng L, Li C, Ji Q, Li P. Salidroside Alleviates Cartilage Degeneration Through NF-κB Pathway in Osteoarthritis Rats. *Drug Des Devel Ther*. 2020;14:1445-1454. https://doi.org/10.2147/DDDT.S242862

● Figure 5A appears to be reused as Figure 5A in:

Chen H, Shi Z, Xing Y, Li X, Fu F. Fangchinoline attenuates cardiac dysfunction in rats with endotoxemia via the inhibition of ERK1/2 and NF-κB p65 phosphorylation. *Ann Transl Med* 2020;8(18):1167. doi: 10.21037/atm-20-5669

● Figure 5A appears to be reused as Figure 6B in:

Xu, M., Li, X., & Song, L. (2020). Baicalin regulates macrophages polarization and alleviates myocardial ischaemia/reperfusion injury via inhibiting JAK/STAT pathway. *Pharmaceutical Biology*, 58(1), 655–663. https://doi.org/10.1080/13880209.2020.1779318

In January 2025, the authors contacted the journal to request withdrawal of their manuscript due to errors in the grouping of the images. When the authors were asked for an explanation regarding the above concerns raised, whilst they cooperated fully, they were unable to address the concerns raised.

As verifying the validity of published work is core to the integrity of the scholarly record, we are therefore retracting the article. The corresponding author listed in this publication has been informed. The author(s) agree with the retraction.

We have been informed in our decision-making by our editorial policies and the COPE guidelines. The retracted articles will remain online to maintain the scholarly record, but they will be digitally watermarked on each page as ‘Retracted’.

